# *Lentinus edodes* Powder Improves the Quality of Wheat Flour Gluten Sticks

**DOI:** 10.3390/foods12091755

**Published:** 2023-04-23

**Authors:** Suya Xie, Hongbo Li, Na Li, Zhenbin Liu, Dan Xu, Liangbin Hu, Haizhen Mo

**Affiliations:** 1 School of Food and Biological Engineering, Shanxi University of Science and Technology, Xi’an 710021, China; 2 College of Food Science and Engineering, Central South University of Forestry and Technology, Changsha 410004, China

**Keywords:** gluten sticks, *L. edodes* powder, wheat flour, extrusion puffing

## Abstract

Spicy wheat flour gluten sticks are delicious and affordable puffed snacks for young adults and even minors in China, and have a relatively simple nutritional quality. *L. edodes* powder (LEP) is rich in nutrients and boasts a variety of biological activities. This study evaluated the effects of different concentrations of LEP addition on the quality of wheat flour gluten sticks. The gelatinization results of the products showed that the peak viscosity decreased from 454 cP to 251 cP; the breakdown value decreased from 169 cP to 96 cP; and the setback value decreased from 381 cP to 211 cP. With the increase in LEP, the radial expansion rate (RER) of *L. edodes* gluten sticks (LSGS) first increased and then decreased, reaching a maximum value of 1.388 in the 10% LEP group. The oil absorption rate (OAR) of LSGS increased from 5.124% to 14.852% with the increase in the amount of LEP. Additionally, texture profile analysis showed that the hardness value increased from 1148.898 to 2055.492 g; the chewiness value increased from 1010.393 to 1499.233; and the springiness value decreased from 1.055 to 0.612. Through X-ray diffraction (XRD), it was found that the crystal type was transformed from A-type crystal to B-type and V-type crystals. Low field nuclear magnetic resonance (LF-NMR) results showed that the moisture distribution in the products was basically bound water. The scanning electron microscopy (SEM) results showed that, with the increase in the LEP amount, the surface of the products changed from rough to smooth. Sensory evaluation results indicated that the products with 10% LEP helped to maintain better taste and quality of LSGS, with an average score of 7.628, which was the most popular among consumers. This study not only increases the possible raw materials for use in extruded puffed food, but also provides a new possibility for the production of high-quality edible fungi extruded products.

## 1. Introduction

Extrusion technology is a high-temperature, short-time food processing method that integrates heating, sterilization, extrusion, and molding [1]. It is one of the high-tech food processing technologies of the 21st century. Extrusion technology has many incomparable advantages over other processing methods [2]. Firstly, starch, protein, and fat such as macromolecules in the foods degrade during extrusion, making them more easily digestible and absorbable by the human body. Secondly, puffing can enhance the taste and flavor of a product. Thirdly, a rich variety of raw materials are available for extrusion. Finally, the processing cost is relatively low. Because of these advantages, extrusion is favored by researchers from all over the world.

As a commercial product, puffed snack food has many advantages, such as good flavor and taste, high digestibility and absorption, low nutritional loss, and easy storage, which makes it deeply loved by consumers. Traditional puffed products used cereal products as the main ingredients, making them rich in a single nutritional component (carbohydrates) with high calorie content. With the development and rapid upgrading of the industry, as well as the continuous improvement of people’s quality of life, the demand for leisure puffed foods has shifted to healthy alternatives. As consumers are seeking not only delicious snack food but also low-calorie, healthy, and nutritious snack food, many food companies in China and abroad have been working on the development of low-fat, low-calorie, low-sugar, and high-protein snack foods as the new direction for puffed snack food industries.

Nowadays, nutritional supplements are added to the traditional ingredients to enrich the nutritional value of puffed products. Many studies on snack products have shown higher nutritional properties and better quality by adding different types of ingredients, such as sesame seed powder [3], soy protein isolate [4], soy flour [5], shrimp powder [6], tomato powder [7], soy flour and oat bran [8], yam powder [9], and vitamins [10]. However, there are limited reports on puffed snack foods with added *Lentinus edodes*. As food-drug homologous fungi, *L. edodes* have antifungal/antibacterial, antiviral, immunomodulatory, and antitumor activities [11]. As foodstuff, *L. edodes* are appetizing and nourishing. Moreover, they are rich in nutrients, including complex carbohydrates, proteins (20–23%) and essential amino acids, and a variety of vitamins and minerals; however, they are low in fat (3–4%) with a high percentage of polyunsaturated fatty acids. The protein in *L. edodes* is composed of 18 types of amino acids, including all essential amino acids in ratios that are similar to those ideal for human nutrition. Additionally, *L. edodes* is rich in trace elements; an appropriate amount of trace elements enhances physical fitness and prevents diseases [12,13,14].

The purpose of this study was to develop a puffed snack with a high nutritional value using *L. edodes* powder (LEP) as the main raw material through a low-cost extrusion processing technology. The feasibility of the puffed snack was evaluated by measuring the nutritional composition, radial expansion rate, oil absorption, gelatinization properties, texture properties, crystal structure, moisture distribution, morphological changes, and sensory evaluation. It is expected that LEP can improve the quality of traditional gluten sticks and provide new ideas for the development of new edible fungi products.

## 2. Materials and Methods

### 2.1. Materials

The wheat flour used to produce LSGS was purchased from Cofco International Beijing Co., Ltd. (Beijing, China). Shiitake mushroom (*Lentiluna edodes*) was purchased from Yipin Fresh Supermarket (Xi*’*an, China). After natural drying, they were pulverized into *L. edodes* powder (LEP; ground and pass through an 80-mesh sieve) by a crusher (RS-FS553, Hefei Rongshida Rong Brand Electric Co., Ltd., Hefei, China).

### 2.2. Nutrient Composition Analysis

Crude protein content was determined by Kjeldahl method (K1160, Shandong Green Carrey Precision Instrument Co., Ltd., Heze, China) [15]. In the sample, copper sulfate, potassium sulfate, and concentrated sulfuric acid were added successively before digestion and distillation. The samples, after alkalization distillation, were immediately titrated by a standard acid. The measured nitrogen content was converted into crude protein content by a conversion factor of 6.25.

Crude fat content was extracted by Soxhlet extraction method (Soxtec2050, Shanghai Ruifen International Trade Co., Ltd., Shanghai, China) [16]. The samples were wrapped within filter paper before being inserted at the bottom of the extraction tube to make them completely immersed in anhydrous ether. Heating was continued in a constant temperature water bath at 70–80 °C. The crude fat content was calculated after drying and weighing.

Moisture content was determined by direct drying method. The weighted samples were dried in an oven (DHG-9245AE, Shanghai Shengshihuike Testing Equipment Co., Ltd., Shanghai, China) at 105 °C for about 12 h until constant weight and moisture content were calculated from the weight difference.

For the determination of total ash, firstly, the weighed samples were heated over a small fire to make them completely carbonized and smokeless. Then, they were put in a muffle furnace at 600 °C (HP-1000W, Shanghai Xiangbei Industry Co., Ltd., Shanghai, China) for ashing for 4 h. After natural cooling below 200 °C, the samples were placed in a dryer for 40 min to cool to room temperature. They were again put into the high temperature furnace for continuous burning for 30 min. After cooling, they were weighed and recorded [17].

Crude fiber content was determined by filtration method according to Lu et al. [18]. Total soluble sugars were assayed by phenol sulfuric acid method [19]. Starch content was determined by enzymatic hydrolysis [20].

### 2.3. Gelatinization Properties

The reconstituted *L. edodes* flour was made by mixing wheat flour with different amounts of LEP (0%, 3.3%, 6.6%, 10%, 13.3%, 16.6%, and 20%). Then, deionized water was added to the *L. edodes* flour at a ratio of 3:1 (*w*/*v*) and transferred to an aluminum canister. Using a rapid visco analyzer (RVA 5280, Anhui Hongzhong Medical Instrument Co., Ltd., Hefei, China), the gelatinization characteristics of the samples were analyzed. The gelatinization properties were calculated according to the method of Wang et al. [21]. The test procedure of RVA was as follows: The mixture samples were heated from room temperature to 95 °C at a rate of 5 °C/min, and then held at 95 °C for 4 min. After that, they were cooled to 50 °C at a rate of 5 °C/min and held for 4 min to obtain the gelatinization curves of different samples.

### 2.4. Extrusion Process

The amount of LEP added was 0%, 3.3%, 6.6%, 10%, 13.3%, 16.6%, and 20%. The proportion of LEP and wheat flour to water was 3:1. LEP, wheat flour, and deionized water in different proportions were mixed evenly by a dough mixer (AM-CG108-1, Hebei Nordic Sino Food Machinery Co., Ltd., Hengshui, China) to form *L. edodes*-based dough. Using a single-screw laboratory extruder (Model 60-001, Jining Mengheng E-Commerce Co., Ltd., Jining, China) with a 20 mm circular die, *L. edodes* gluten sticks (LSGS) were prepared by extrusion at high temperature. The feeding rate was adjusted from 400 g/min to 800 g/min and stabilized. The extruded LSGS were then cut off, after which they were cooled to room temperature for 2 min, then stored in a sealed PE bag at 25 °C until they were tested.

In addition, 30 samples were retained for each group of extruded products with different LEP additions and each group of experiments was conducted three times during the experiment.

### 2.5. Radical Expansion Rate (RER) and Oil Absorption Rate (OAR)

The radial expansion rate (RER) and oil absorption rate (OAR) of LSGS were determined by referring to previous studies [22]. Ten randomly selected LSGS were measure for their diameters using a Vernier caliper to take the average diameter, D. The diameter of the mould was measured several times (d). The RER was calculated according to the equation below:

(1) $$RER(\%)=\frac{D}{d}\times 100$$

The weighted LSGS (m_1_) were cut into 3 sections before being placed in a 50 mL centrifuge tube filled with vegetable oil. After vortexing for 1 min, they were left at room temperature for 30 min. The samples were gently patted with filter paper to absorb the surface oil. After centrifugation for 5 min, the mass (m_2_) was recorded. This procedure was repeated 3 times to obtain the average value. The OAR was calculated by the equation below:

(2) $$OAR(\%)=\frac{m_{2}-m_{1}}{m_{1}}\times 100$$

### 2.6. Texture Profile Analysis (TPA)

Referring to the method described by Jia et al., with some modifications [23], the texture properties of the samples before and after extrusion were studied by using a texture analyzer (TX-XT Plus, Beijing Oriental Anno Technology Co., Ltd., Beijing, China). The hardness and chewiness were determined in full texture mode using a cylindrical probe (P/75) with a diameter of 75 mm. During the TPA test, two compression cycles were performed. After the first compression was completed, the plunger was reversed at 2 mm/s and held for 5 s to begin the second compression. The test procedure was as follows: Pre-test speed of 5 mm/s; test speed of 2 mm/s; and post-test speed 2 mm/s with a compression strain of 45% and trigger force of 10 g.

### 2.7. X-ray Diffraction Pattern (XRD)

The change in starch crystallinity in the materials before and after extrusion was measured by an X-ray diffractometer (D8 Advance, Bruker AXS Inc., Karlsruhe, Germany). The sample scanning method was based on Li et al. [24], with slight modification. The extruded products were freeze-dried using a vacuum freeze dryer (ALpHA2-4 LD plus, Marin Christ., Osterode, Germany) and then ground to powder (to pass through 80-mesh sieve after grinding). The scanning voltage was 45 kV; the current was 40 mA; the diffraction angle (2θ) was between 4° and 50°; and the scanning rate was 2° per min.

### 2.8. Low Field Nuclear Magnetic Resonance (LF-NMR)

A certain length of extruded LSGS was weighed and placed in a NMR tube. A low-field nuclear magnetic resonance (VTMR20-010V-1, Suzhou Newmai Analytical Instrument Co., Ltd., Suzhou, China) was used to measure moisture migration and distribution in the samples [25]. During the experiment, the transverse relaxation time (T_2_) of the sample was measured by CPMG pulse sequence. The test parameters of CPMG sequence were set as follows: 90° pulse time (P1) was 7 μs; 180° pulse time (P2) was 14 μs; number of echoes (NECH) was 18,000; echo time was 0.8 ms; accumulation times were conducted 8 times; sampling point (TD) was 59,992; spectral width (SW) was 200 kHz; and sampling repetition time (TW) was 3000 ms. The instrument software was used to invert the obtained data and the T_21_, T_22_, and T_23_ parameters were fitted. Three replications were made.

### 2.9. Scanning Electron Microscope (SEM)

The samples were placed in a −80 °C refrigerator and precooled for 24 h. LSGS were dried by a vacuum freeze dryer (ALpHA2-4 LD plus, Marin Christ., Osterode, Germany). After lyophilization, the materials were cut into thin slices with a stainless-steel blade. They were then adhered to the sample holder with conductive double-sided tape and the excess powder was blown off with an ear-washing ball. After spraying gold on the sample, the microstructure was scanned and observed under a field emission scanning electron microscope (Hitachi SU8010, Hitachi Limited, Tokyo, Japan) (5–10 kV) with an accelerating voltage and at a magnification of 2500 times. Ten pictures were taken from each sample.

### 2.10. Sensory Evaluation

Sensory evaluation was performed at Shaanxi University of Science and Technology, Xi’an, Shaanxi, China on the next day of the production of LSGS. Altogether, 20 evaluators were selected who had all participated in the food sensory course training to conduct sensory evaluation, and the average value was calculated. The samples were randomly numbered before the test. There was a certain time interval between the evaluations of each sample. No communication between the evaluators was allowed. In the sensory evaluation, after tasting each sample, they were asked to gargle their mouths with water in order to prevent “taste masking”. The evaluators were asked to use the 9-point hedonic scale from 1 to 9 to evaluate the product and to evaluate the following attributes of the sample: Color, odor, chewiness, elasticity, and apparent structure of the products [26], where 1 means “extremely dislike” and 9 means “extremely like”.

### 2.11. Statistical Analysis

Unless otherwise specified, all measurements were made in triplicate. SPSS analysis software (SPSS Statistics 17.0, SPSS Inc., Chicago, IL, USA) was applied to analyze data and Origin 9.0 (OriginLab Corp., Northampton, MA, USA) was used for data processing and graphic construction. Difference of *p* < 0.05 was judged to be significant using Duncan’s test.

## 3. Results and Discussion

### 3.1. Nutrient Composition Analysis

The main nutritional components in the LEP and wheat flour are shown in Table 1. It can be seen that the total sugar content of the LEP was 52%; protein content was 21.2%; and the contents of water, crude fiber, total ash, and crude fat were 8.6%, 7.5%, 4.9%, and 2.9%, respectively. However, the wheat flour mainly contained 68% starch and 9.5% protein, while the contents of water, crude fiber, crude fat, and total ash were 10.5%, 2.1%, 1.5%, and 1.2%, respectively. The contents of protein, crude fiber, crude fat, and total ash in the LEP were high. Therefore, the addition of LEP to wheat flour increased the proportion of protein, crude fiber, crude fat, and minerals in the mixture, while it reduced the proportion of carbohydrates, ultimately improving the nutritional qualities of the flour products.

**Figure 1:**
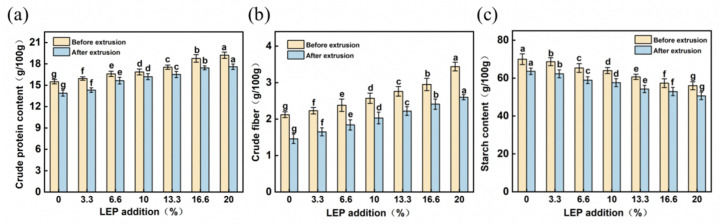
Changes in (**a**) crude protein; (**b**) crude fiber; and (**c**) starch content in samples before and after extrusion. Each error bar indicates mean ± SD. Different letters in same color bars are statistically different between the treatments (*p* < 0.05).

We can see from Figure 1 that, with the increase in the LEP dosage, the proportion of protein, crude fiber, and crude fat in the mixture gradually increased, while the proportion of starch gradually decreased. However, after the extrusion process, the content of nutrients in the mixed materials, including crude protein, crude fiber, and starch, decreased slightly compared with those before extrusion. This was mainly because protein molecules might undergo hydrolysis during extrusion, and protein macromolecules might be converted into small amino acid molecules, making them easier for human absorption. The degradation of the wheat starch occurred in the form of a mixture of gelatinized starch, molten starch, and degraded starch, resulting in a decrease in starch content [27]. The decrease in crude fiber content might be due to the fact that, when the water content was low, the material was subjected to a greater shear force in the barrel, which caused more destruction of the insoluble dietary fiber, leading to the decrease in crude fiber content [28].

### 3.2. Gelatinization Properties

**Figure 2:**
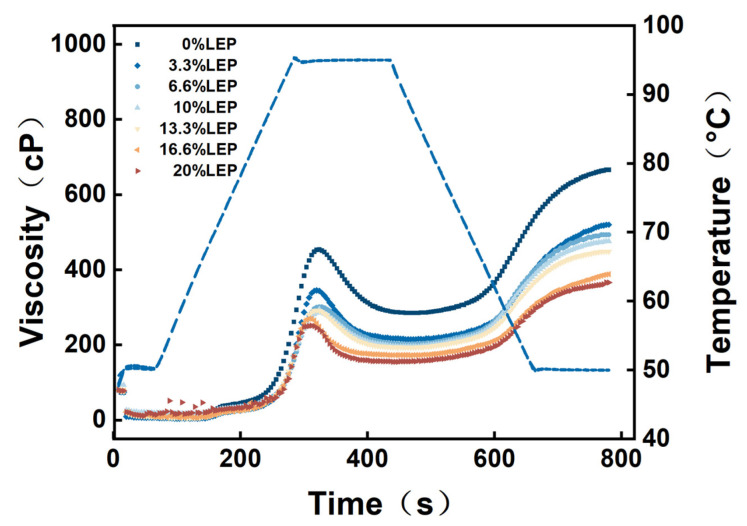
The gelatinization curves of wheat flour with different concentrations of LEP (0, 3.3%, 6.6%, 10%, 13.3%, 16.6%, and 20%, wt).

The gelatinization characteristic curves of wheat flour with different concentrations of LEP are shown in Figure 2. Obviously, with the increase in the amount of LEP added, the gelatinization behavior of the samples showed clear differences. The gelatinization characteristic parameters obtained from the gelatinization characteristic curves of the different LEP dosages are shown in Table 2. We found that the addition of different doses of LEP resulted in significant reductions in peak, trough, and final viscosities, as well as breakdown and setback values.

Gelatinization parameters are of great significance for recognizing the sensory properties of modified starch and its potential industrial prospects [29]. Peak viscosity (PV), which reflects the swelling ability of starch, was generated by friction after the starch particles expanded with sufficient water absorption [30], and this was due to the weakening of the internal structure of the particles [31]. It can be seen that PV showed a gradually decreasing trend, from 454 ± 8.12 cP to 251 ± 7.44 cP (Table 2). The decrease in PV might mean that LEP could cover the wheat starch granules to prevent them from swelling in free water. This finding indicates that LEP affects the swelling property of wheat starch granules, leading to a decrease in PV [32].

The breakdown value represents the difference between the peak viscosity and trough viscosity, reflecting the degree of destruction of starch granules during heating and the stability of starch paste [21]. The breakdown value of the wheat flour–LEP paste was reduced from 169 ± 6.71 cP to 96 ± 6.63 cP with the addition of LEP from 0% to 20%, suggesting that LEP was able to inhibit the breakage of wheat starch granules and enhance the stability of hot paste. The setback value represented the difference between the final and trough viscosities, which reflected the increase in viscosity of the starch paste during RVA cooling. At the same time, the setback value decreased from 381 ± 8.42 cP to 211 ± 6.58 cP as the amount of LEP increased, and the decrease in setback might be due to the steric hindrance caused by the introduction of hydrophobic groups, which prevented the close packing of starch chains after cooling [29]. These results indicated that LEP could restrain the short-term retrogradation of wheat flour by replacing part of the starch, indicating that LEP was able to significantly change the gelatinization characteristics of wheat flour. This might be because LEP can preserve water in wheat starch granules, which reduced the initial expansion of starch granules.

### 3.3. Extruded Materials

**Figure 3:**
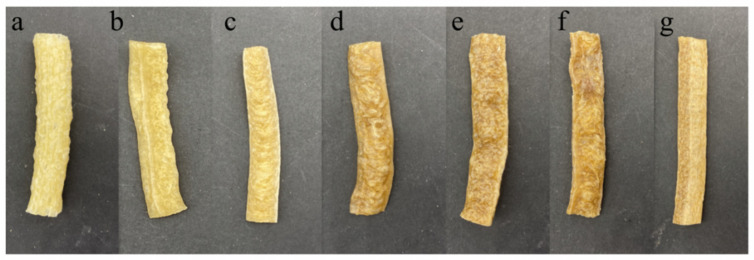
LSGS extruded products diagram with different concentrations of LEP: (**a**) 0%; (**b**) 3.3%; (**c**) 6.6%; (**d**) 10%; (**e**) 13.3%; (**f**) 16.6%; and (**g**) 20%.

Figure 3 is the extrusion effect diagram of the materials when adding different ratios of LEP. We can see that, with the increase in the amount of LEP addition, the materials exhibited different puffing effects after extrusion. When only wheat flour was added, the colors of the extruded materials were the brightest and the products showed a better puffing effect. When trace amounts (3.3% and 6.6%) of LEP were added, the extruded products of LSGS were light-colored; the extruded products were soft; and there was no significant difference in the puffing effect compared with the control group. When an appropriate amount (10% and 13.3%) of LEP was added, the surface was shiny and the products were light brown with uniform small holes inside, suggesting that the composite system of wheat flour–LEP had a better puffing effect. When a large amount (16.6% and 20%) of LEP was added, the extruded products were dark brown, resulting in a worse puffing effect, and the extruded products were hard. When the added amount of LEP was 16.6%, the surface of the product was extremely uneven and when the amount of LEP was 20%, the puffing effect was almost zero and the color of the products was not uniform.

### 3.4. Radial Expansion Rate (RER) and Oil Absorption Rate (OAR)

The RER and OAR are two important indexes for evaluating the quality of LSGS products. The RER characterizes the increase in the cross-section diameter of the product obtained through the extruder die head, as compared to the diameter of the die head. The larger the ratio, the higher the expansion degree. The OAR represents the ability of the product to retain oil after adsorption. The better the oil holding capacity of the material, the stronger the oil adsorption on LSGS; however, a higher OAR can cause a greasy feeling when eating.

Table 3 shows that, compared with the control group, adding different proportions of LEP had a significant effect on the RER and OAR of the LSGS. With the increase in the amount of LEP, the RER showed a trend of slightly rising at first and then declining. It can be seen that the RER of the LSGS reached 1.131 when no LEP was added, and then gradually increased to 1.264 when a small amount (6.6%) of LEP was added. When the amount of LEP added reached 10%, the RER reached the highest value of 1.388. When more than 10% LEP was added, the RER of the products continued to decrease. We found that mixing a certain proportion of LEP can improve the RER of LSGS; this might be related to the fact that different materials change their structure after heating. After adding LEP, the degree of cold shrinkage of the LSGS decreased during the process of leaving the mould, wheat starch granules crystallized in a molten state during extrusion, the tension required for its expansion was greater than that of the LEP, and the wheat starch granules were less likely to break causing the RER of the mixture of LEP and wheat flour to increase [33]. When the addition amount of LEP reached 20%, the RER decreased to 0.833, which might be because (compared with wheat flour) the LEP more easily absorbed the moisture. Excessive (13.3%, 16.6%, and 20%) addition of LEP might dilute the starch content in the mixture, resulting in an incomplete puffing of the extruded product. In this way, the RER of the LSGS during the extrusion and puffing process was reduced. In addition, when the amount of LEP was increased, the OAR of the products gradually increased, which might be because the LEP had a certain encapsulation effect on the oil.

### 3.5. Texture Profile Analysis (TPA)

Texture analysis is a method for evaluating the physical properties, mechanical properties, and product quality of materials. For puffed foods, hardness, chewiness, and springiness, collectively, reflect their taste and texture. When the hardness and chewiness of the extruded products were too small, the overall structure was too soft, resulting in a lack of toughness during chewing. When the hardness and chewiness were too high, the phenomenon of “slagging” was more likely to occur in the later storage process of the extruded products. Table 4 shows that, with the increase in the amount of LEP, the hardness and chewiness of the products significantly increased, while the springiness presented a gradual downward trend. This was related to the texture characteristics of LEP itself, and the cellulose content in the LEP was high. Therefore, with the increase in the amount of LEP, the chewiness and hardness of the extruded products increased. Previous texture analysis studies have shown a significant positive correlation between hardness and chewiness [34]. In addition, we found that adding too much LEP might have a certain negative impact on product quality. High concentrations (20%) of LEP resulted in a reduced hardness and chewiness of the extruded products. Appropriate concentrations (6.6–10%) of LEP were able to render the taste of the product more chewy, with less hardness and moderate springiness, which was more suitable for those who enjoy a chewy texture. When the concentration of LEP was low (3.3%), the product was more elastic, but their hardness and chewiness were lower, and they tend to be softer during chewing.

### 3.6. X-ray Diffraction Pattern (XRD)

**Figure 4:**
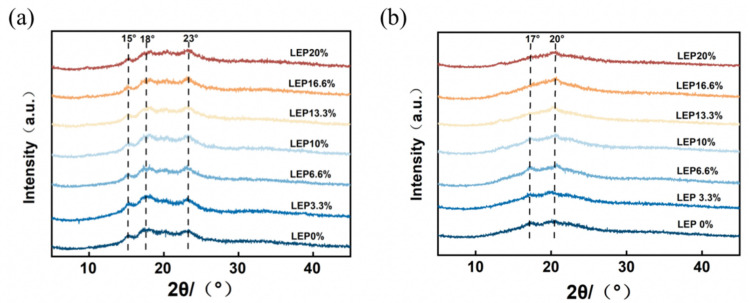
X-ray diffraction of wheat flour dough with different concentrations of LEP. (**a**) Mixed materials before extrusion and (**b**) LSGS extruded products.

XRD is the main method used to study crystal structures and their change law, which showed the change of the semi-crystalline structure in starch during esterification [29]. X-ray diffractograms of the LSGS are displayed in Figure 4. It can be seen from Figure 4a that the wheat flour before gelatinization had diffraction peaks at 15°, 18°, and 23°, indicating that the wheat flour was a typical A-type pattern, which was consistent with previous results [35]. After adding different proportions of LEP, no new diffraction peaks appeared among different products. This showed that the amount of LEP addition had no effect on the crystallinity of the materials. In Figure 4b, extrusion treatment altered the structure of the wheat starch. After gelatinization, the original diffraction peaks at 15°, 18°, and 23° in wheat flour disappeared, while new diffraction peaks appeared near 17° and 20°; the intensity was weak. From these results, it can be speculated that the extruded wheat flour changed from A-type to B-type and V-type crystals [36]. The changes in crystal structure were probably due to the intramolecular and intermolecular hydrogen bonds being broken after the wheat flour was extruded and expanded, and the double helix structure was disintegrated and the wheat starch granules were broken, which further destroyed the crystalline structure of the wheat starch, making the original crystalline region into an amorphous region. However, after adding different ratios of LEP, the diffraction peak intensity around 17° gradually weakened until it disappeared with the increase in the LEP addition. It was explained that the amount of LEP added had an effect on the internal crystal structure of the LSGS. In addition, the peak was shifted to around 20°, which was a typical diffraction peak of amylose-lipid complexes. The complex might hinder the rearrangement of amylose and delay the occurrence of retrogradation [37].

### 3.7. Low Field Nuclear Magnetic Resonance (LF-NMR)

**Figure 5:**
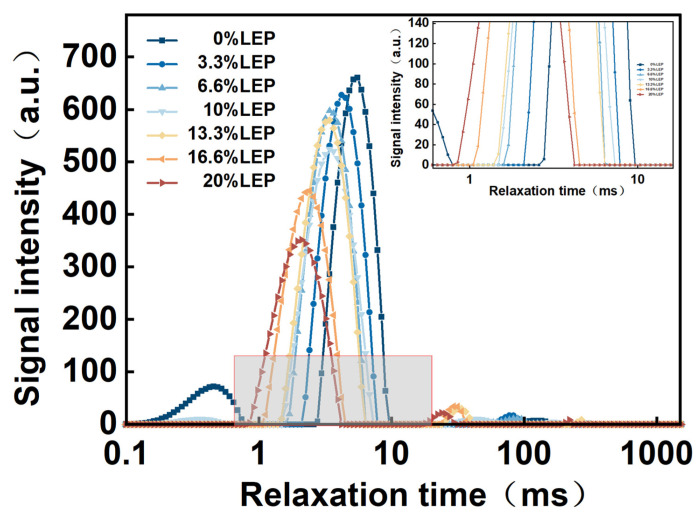
The transverse relaxation time (T_2_) curves of LEP (0, 3.3%, 6.6%, 10%, 13.3%, 16.6, and 20%, wt) on LSGS extruded products.

Figure 5 shows the relaxation time distribution curves of the LSGS samples at different times. In the inversion atlas, we can see that there were three peaks; from left to right, they were T_21_ (0.1–10 ms), T_22_ (10–100 ms), and T_23_ (100–1000 ms). The relaxation time T_21_ water population, with relatively concentrated relaxation times, was defined as “bound water”, accounting for the maximum proportion, which represented the water fraction that is tightly bound to other less mobile molecules. T_22_ was defined as “semi-bonded water”, and this portion of the water was bound by a certain binding force and did not flow easily. The relaxation time T_23_ water population, with the longest relaxation time being greater than 100 ms, had the molecular mobility of water in an aqueous solution, indicating that the most fluid part is the free water part [38]. The shorter the relaxation time, the smaller the degree of freedom of water and the tighter the combination with the material.

The peak area reflected the water content under different conditions; with the addition of LEP, both the area of T_2_ and each peak changed greatly. As can be seen from Figure 5, the changes in moisture content also showed a similar trend. Firstly, the peak area gradually decreased and, secondly, the peak moved to the left [25]. The moisture distribution in the extruded products was basically composed of bound water, low levels of semi-bonded water, and free water. After extrusion, the wheat starch was more likely to form hydrogen bonds with water; therefore, that the water in the starch granules increased, resulting in an increase in the bound water content. With the increase in the amount of LEP, the T_21_ relaxation time was significantly shortened to a shorter relaxation time and the T_22_ and T_23_ relaxation times did not change significantly, indicating that the addition of LEP caused the water in the LSGS to be in a less fluid state.

### 3.8. Scanning Electron Microscope (SEM)

**Figure 6:**
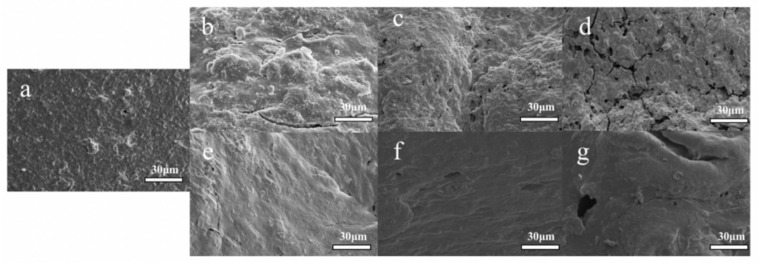
SEM images of LSGS extruded products with different concentrations of LEP: (**a**) 0%; (**b**) 3.3%; (**c**) 6.6%; (**d**) 10%; (**e**) 13.3%; (**f**) 16.6%; and (**g**) 20%.

The SEM images of the LSGS samples with different concentrations of LEP are shown in Figure 6. It can be observed that the starch particles and proteins on the surface of the freeze-dried samples form dense aggregates and different concentrations of LEP can affect the surface structure of LSGS. This might be due to the gelatinization of the wheat starch, fiber degradation, and protein denaturation at a high temperature, high pressure, and high shear environment inside the barrel. In addition, compared to the control group, with the increase in the amount of LEP, the surface of the LSGS first became rough and porous, and then became relatively smooth. Many starch particles appeared on the surface of the control group products. When the amount of LEP addition was 3.3% and 6.6%, the surface of the LSGS was uneven, and a large number of protrusions could be seen on the product surface. When the amount of LEP addition was 6.6%, some scattered tiny holes could be seen on the material surface. When the amount of LEP addition was 10%, the pores on the surface of the product became larger and evenly distributed among the materials, leading to significant fracture cracks. At this time, the expansion effect of the material reached the highest point, which was also consistent with the previous RER measurement results. Only the samples with 6.6% and 10% LEP addition had obvious pore-like structures on the surface. With the increase in the amount of LEP addition (when the amount reached 13.3%, 16.6%, and 20%), the surface of the extruded sample became relatively smooth and the internal structure became denser. SEM analysis also further demonstrated that the protein molecules of LEP can enhance their binding to starch in wheat flour through hydrogen bonds. As a result, the surface of the LSGS became smoother, which was similar to previous research results [23].

### 3.9. Sensory Evaluation

**Figure 7:**
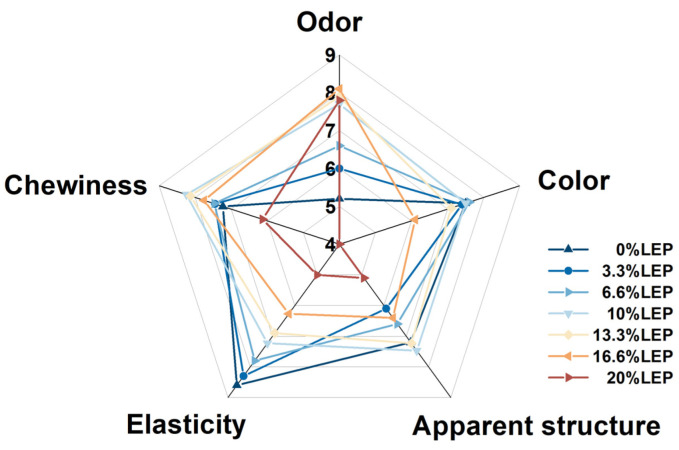
Sensory evaluation of LSGS extruded products with different concentrations of LEP (0, 3.3%, 6.6%, 10%, 13.3%, 16.6, and 20%, wt). The higher the numerical value, the higher the degree of liking, 4–6 represented “generally like”, 7–9 represented “extremely like”.

The sensory evaluation results of LSGS with different LEP additions are shown in Figure 7. The odor and chewiness scores of the LSGS showed a trend of first increasing and then decreasing with the increase in LEP addition. When the LEP addition was 16.6%, the product had a strong odor, with the highest score reached being 8.1. When the amount of LEP addition was 6.6–16.6%, the product had no impurities, no hard lumps, and good chewiness; the products with 10% and 13.3% LEP scored the highest. When the amount of LEP addition was too high (16.6% and 20%), the surface color of the LSGS was uneven and the color score significantly decreased. When the amount of LEP addition was less than 16.6%, there was no significant difference in the color score of the products. The apparent structure is characterized by a uniformity of the pores on the cross-section of the LSGS. The results showed that the pores on the cross-section of the product with 10% and 13.3% LEP additions were small and uniform, and the scores were high. When the amount of LEP addition was too high (20%), due to its small expansion rate, there were few pores in the cross-section and the distribution was uneven, with a significantly lower score than that of the other samples. The elasticity score of the LSGS gradually decreased with the increase in the amount of LEP addition. Based on the five indicators of odor, color, chewiness, elasticity, and apparent structure, when the amount of LEP added reached 10%, the comprehensive score was the highest, with an average of 7.628, which was the most popular among consumers.

## 4. Conclusions

This study explored the feasibility of preparing gluten sticks using *L. edodes* powder as raw material through extrusion. With the increase in the amount of LEP addition, the peak viscosity, breakdown value, and setback values decreased. The oil absorption rate of the products gradually increased, and the expansion rate showed a trend of increasing first and then decreasing, with the highest expansion rate being 1.388 for products with 10% LEP addition. The hardness and chewiness of the LSGS increased and the springiness decreased. After extrusion, the samples changed from A-type crystals to B-type and V-type crystals. The water content in the LSGS was basically bound water and the bound water content gradually decreased with the increase in LEP addition; with the increase in the amount of LEP addition, the surface of the products changed from rough to smooth. In addition, the highest comprehensive score for LSGS with 10% LEP addition was 7.628, which was deeply loved by consumers. This study showed a good application potential of *L. edodes* in the preparation of highly nutritious foods by extrusion and provided a new possibility for expanding the application of edible fungi.

## Figures and Tables

**Table 1:** The main components of wheat flour and LEP.

Component	Crude Protein	Crude Fat	Crude Fiber	Water	Total Sugars	Total Ash	Starch
LEP	21.2 ± 1.56 ^a^	2.9 ± 0.22 ^a^	7.5 ± 0.68 ^a^	8.6 ± 0.81 ^a^	52 ± 5.72	4.9 ± 0.53 ^a^	No measured
Wheat flour	9.5 ± 0.95 ^b^	1.5 ± 0.11 ^b^	2.1 ± 0.19 ^b^	10.5 ± 0.98 ^a^	No measured	1.2 ± 0.23 ^b^	68 ± 5.23

NOTE: Each value indicates mean ± standard deviation. Using the *t* test, different letters in the same column showed significant difference (*p* < 0.05).

**Table 2:** Gelatinization parameters with different concentrations of LEP (0, 3.3%, 6.6%, 10%, 13.3%, 16.6, and 20%, wt).

Samples ^a^	Viscosity (cP)				
	Peak	Trough	Final	Breakdown	Setback
0%	454 ± 8.12 ^a^	285 ± 7.84 ^a^	666 ± 9.52 ^a^	169 ± 6.71 ^a^	381 ± 8.42 ^a^
3.3%	345 ± 7.63 ^b^	215 ± 9.02 ^b^	520 ± 6.81 ^b^	130 ± 7.23 ^b^	305 ± 9.07 ^b^
6.6%	301 ± 6.68 ^c^	201 ± 8.61 ^c^	493 ± 7.95 ^c^	100 ± 9.86 ^c^	292 ± 7.41 ^c^
10%	295 ± 9.17 ^d^	196 ± 6.73 ^d^	476 ± 8.69 ^d^	99 ± 5.41 ^c^	280 ± 7.80 ^d^
13.3%	292 ± 5.93 ^e^	193 ± 5.89 ^e^	449 ± 9.36 ^e^	99 ± 7.04 ^c^	256 ± 8.53 ^e^
16.6%	270 ± 9.16 ^f^	173 ± 8.05 ^f^	388 ± 6.82 ^f^	97 ± 9.36 ^d^	215 ± 5.94 ^f^
20%	251 ± 7.44 ^g^	155 ± 9.08 ^g^	366 ± 8.97 ^g^	96 ± 6.63 ^d^	211 ± 6.58 ^g^

NOTE: The percentage numbers denoted the percentages of LEP concentration contained in the samples. Each value indicates mean ± standard deviation. Different letters in the same column showed significant difference (*p* < 0.05).

**Table 3:** Effect of different concentrations of LEP (0, 3.3%, 6.6%, 10%, 13.3%, 16.6, and 20%, wt) on the RER and OAR of LSGS extruded products.

Samples	RER	OAR (%)
0%	1.131 ± 0.0173 ^d^	5.124 ± 1.2003 ^f^
3.3%	1.189 ± 0.0507 ^c^	5.291 ± 0.9346 ^f^
6.6%	1.264 ± 0.0287 ^b^	5.673 ± 0.7577 ^e^
10%	1.388 ± 0.0289 ^a^	12.436 ± 0.6744 ^d^
13.3%	1.128 ± 0.0326 ^d^	13.917 ± 1.1726 ^c^
16.6%	0.974 ± 0.0533 ^e^	14.156 ± 1.4752 ^b^
20%	0.833 ± 0.0243 ^f^	14.852 ± 1.0925 ^a^

NOTE: The percentage numbers denote the percentages of LEP concentration contained in the samples. Each value indicates mean ± standard deviation. Different letters in the same column show significant difference (*p* < 0.05).

**Table 4:** Effect of different concentrations of LEP (0, 3.3%, 6.6%, 10%, 13.3%, 16.6, and 20%, wt) on the texture properties in hardness, chewiness, and springiness of LSGS extruded products.

Samples	Hardness (g)	Chewiness	Springiness
0%	1148.898 ± 32.32 ^f^	1010.393 ± 56.28 ^g^	1.055 ± 0.028 ^a^
3.3%	1293.307 ± 24.45 ^e^	1182.01 ± 49.01 ^f^	0.942 ± 0.011 ^b^
6.6%	1488.048 ± 45.38 ^d^	1266.978 ± 61.31 ^e^	0.845 ± 0.014 ^c^
10%	1661.984 ± 26.71 ^c^	1366.23 ± 53.27 ^d^	0.799 ± 0.026 ^d^
13.3%	2055.741 ± 33.9 ^b^	1400.88 ± 42.12 ^c^	0.739 ± 0.021 ^e^
16.6%	2163.826 ± 45.38 ^a^	1565.4 ± 47.35 ^a^	0.702 ± 0.017 ^f^
20%	2055.492 ± 54.59 ^b^	1499.233 ± 32.81 ^b^	0.612 ± 0.031 ^g^

NOTE: The percentage numbers denoted the percentages of LEP concentration contained in the samples. Each value indicated mean ± standard deviation. Different letters in the same column showed significant difference (*p* < 0.05).

## Data Availability

Data is contained within the article.
